# Recovering complete plant root system architectures from soil via X-ray μ-Computed Tomography

**DOI:** 10.1186/1746-4811-9-8

**Published:** 2013-03-20

**Authors:** Stefan Mairhofer, Susan Zappala, Saoirse Tracy, Craig Sturrock, Malcolm John Bennett, Sacha Jon Mooney, Tony Paul Pridmore

**Affiliations:** 1 Centre for Plant Integrative Biology, University of Nottingham, Sutton Bonington Campus, Nottingham, LE12 5RD, UK; 2 School of Computer Science, University of Nottingham, Jubilee Campus, Nottingham, NG8 1BB, UK; 3 School of Biosciences, University of Nottingham, Sutton Bonington Campus, Nottingham, LE12 5RD, UK

**Keywords:** Root systems architecture, 3D, X-ray Computed Tomography, Image analysis, Root phenotyping

## Abstract

**Background:**

X-ray micro-Computed Tomography (μCT) offers the ability to visualise the three-dimensional structure of plant roots growing in their natural environment – soil. Recovery of root architecture descriptions from X-ray CT data is, however, challenging. The X-ray attenuation values of roots and soil overlap, and the attenuation values of root material vary. Any successful root identification method must both explicitly target root material and be able to adapt to local changes in root properties.

RooTrak meets these requirements by combining the level set method with a visual tracking framework and has been shown to be capable of segmenting a variety of plant roots from soil in X-ray μCT images. The approach provides high quality root descriptions, but tracks root systems top to bottom and so omits upward-growing (plagiotropic) branches.

**Results:**

We present an extension to RooTrak which allows it to extract plagiotropic roots. An additional backward-looking step revisits the previous image, marking possible upward-growing roots. These are then tracked, leading to efficient and more complete recovery of the root system. Results show clear improvement in root extraction, without which key architectural traits would be underestimated.

**Conclusions:**

The visual tracking framework adopted in RooTrak provides the focus and flexibility needed to separate roots from soil in X-ray CT imagery and can be extended to detect plagiotropic roots. The extended software tool produces more complete descriptions of plant root structure and supports more accurate computation of architectural traits.

## Background

The way roots develop in soil can have a critical effect on plant growth and impacts crop yield, which is vital to efforts to ensure food security [1,2]. This has prompted the development of a variety of methods for characterising root systems. Many of these require plants to be grown in artificial environments designed to increase the visibility of, and so ease the process of imaging, their roots. Controlled environment methods include hydroponic (Price et. al [3]) and aeroponic (Zobel et. al [4]) techniques, and often rely on artificial growth media such as semitransparent nutrient agar [5,6], gellan gum [7] or transparent soil (Downie et. al [8]). Though image analysis is made more tractable, questions are raised regarding the effect these environments might have on root development.

The most common method used to study the root systems of plants grown in their natural soil environment is root washing [9,10]. This, however, often leads to the underestimation of fine roots through breakage during the washing process. The three-dimensional, spatial distribution of the root system is also lost, limiting the architectural traits that can be recovered. Rhizotrons and minirhizotrons [11]– [13] allow roots to be imaged from within the soil, and have been used extensively, but artificially restrict the direction of root growth to two dimensions. Introduction of the artificial boundary may even affect local soil properties, making conditions near the rhizotron different to those elsewhere in the field, which in turn might impact on root growth. In addition, observations are limited to the boundary surface of the rhizotron, and so reveal only a small fraction of the root architecture.

X-ray micro-Computed Tomography (μCT) provides an attractive alternative. X-ray μCT is a non-destructive imaging technique that can visualize the internal structure of opaque objects. μCT scanners acquire a series of projections from different angles, measuring the attenuation of ionizing radiation passing through the target object. These projections are combined to reconstruct a three-dimensional data set. Data values recorded at each voxel reflect the density of the imaged material and are usually mapped to greyscale intensity values for visualization purposes [14]. μCT is not subject to the constraints facing light-based imaging techniques and enables non-invasive, non-destructive imaging of roots growing in soil.

Though many researchers have shown μCT to be an efficient tool with which to visualize root systems [15], the automatic extraction of quantitative descriptions of root architecture from the resulting data sets presents challenges. First, the X-ray attenuation values of plant roots and the organic matter present in soil overlap. This makes it impossible to specify a root/soil classification criterion based on attenuation alone; some additional information is needed. Heeraman et al. [16] provide that information during a training stage. Here, selected voxels are manually assigned to different classes representing the components contained in the scanned specimen, effectively building a model of the X-ray attenuation data expected from each material. This model is then used to interpret the remainder of the data, separating roots from soil. The approach is, however, sensitive to noise and heavily reliant on the user’s contribution.

A further problem is that the X-ray attenuation values of root material vary, as a result of differential water retention and changes in the density of root material with age. A model of root attenuation built from measurements at the top of the data volume will not be effective at the bottom, and vice versa. This has a profound effect on the performance of threshold-based methods, which have been widely used but require error-correcting post-processing [17]– [21].

Any successful root identification method must both explicitly target root material and be able to adapt to local changes in root properties. Recently, RooTrak [22] adopted a tracking-based strategy, viewing the data volume as a stack of cross-sectional images and following and extracting root objects as they appear to move through them. Visual tracking algorithms build and maintain models of the likely motion and appearance of the target object, using the motion model to predict where the object of interest will appear in the next image and the appearance model to locate it. By adopting a simple motion model [22] capturing the knowledge that roots are connected, RooTrak’s tracking framework focuses analysis on the root. The only user input required is a single mouse click indicating the root in the first image. Updating the appearance model during tracking allows RooTrak to adapt to local changes in root greyscale while distinguishing root from non-root materials with similar intensities.

RooTrak has been shown to be capable of separating root systems from their soil environment in μCT images, and recovering root system architecture traits. The tracking technology upon which RooTrak is based [22] allows the target object to split, allowing the root to branch as it “moves” down the soil column. RooTrak, however, considers image slices in fixed top to bottom order, making upward-growing laterals problematic.

**Figure 1:**
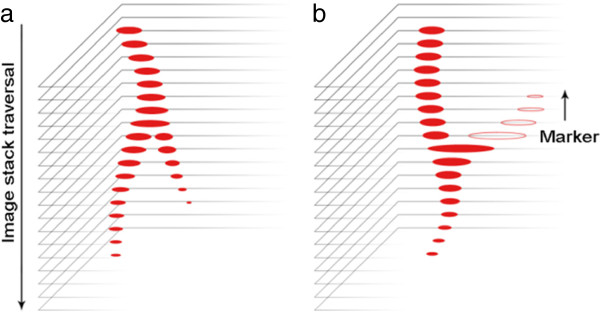
**a) When tracking roots from top to bottom of the image sequence RooTrak’s tracking mechanism allows targets to split, successfully recovering branched architectures. b**) Plagiotropic roots, however, are overlooked. They only appear in the image sequence before they join the primary.

Downward-growing laterals cause the tracked root object to split, so that the initial single root becomes many, each following its own, visible, path through the remainder of the image sequence (Figure 1a). Upward growing (plagiotropic) roots, however, appear before, and not after, their connection to the primary root. Unless they are long enough to appear at the top of the image stack and are marked by the user, RooTrak will simply be unaware of their existence (Figure 1b). The problem is not restricted to root branches, but arises whenever roots grow upwards.

Many root systems contain some roots that grow plagiotropically as they explore the soil for water and nutrients (Nakamoto, [23]. In what follows we describe an extended RooTrak which captures plagiotropic roots, producing more complete root system descriptions and so improved measurement of root system architecture traits.

## Implementation

RooTrak relies upon the level set method [24]. The user marks a single point at the top of the root system in the first image, and a novel variant [22] of the level set approach is applied. The effect is to perform a local segmentation of the image, marking pixels around the start point with sufficiently similar grey values. A connected component algorithm is applied to group adjacent marked pixels together, and the distribution of grey levels within the connected component is recorded. This distribution constitutes a model of the X-ray attenuation of nearby root material.

This first root object is then used to initialise analysis of the next slice (image). It is assumed that the root will appear at a similar position, and with similar size, shape and attenuation properties in the following image. The level set method is initialised with the previous object’s size, shape and position information, and deforms its initial segment description to identify a new region with a similar attenuation distribution. The connected component algorithm is applied again, a new attenuation model computed, and the process continues through the image stack. Care must, however, be taken to ensure that the attenuation model is only updated using reliable root objects. For further details see Mairhofer et al. [22].

When the root branches, the set of pixels identified by the level set becomes separated and the connected component algorithm will identify more than one component. This is a key feature of the level set approach, and one which allows different attenuation models to be associated with, and used to extract, different root branches. RooTrak therefore adapts its attenuation model to suit both different root branches, and to reflect changes in attenuation along each branch. Output is a voxel-based representation of the root, from which a variety of traits are recovered. The tracker, however, only includes root material that is directly connected to a known root object and visible as it proceeds down the stack.

To address this, an additional step has been introduced to RooTrak, allowing it to ‘look back’ for plagiotropic roots. After all root objects have been identified in image n, and before advancing to image n + 1, RooTrak revisits image n-1. This second examination of image n-1 is initialized with the root objects extracted from image n. If the root objects detected when looking back at image n-1 were all found on the forward pass, no plagiotropic roots are present. If, however, additional objects are identified, i.e. more connected components are reported when approaching an image from below than were seen from above, we consider the new objects to be potential upward growing roots, and mark them as such (Figure 1b). Processing then continues downwards (i.e. with image n + 1) until the entire stack has been traversed. The result at this point, following a single completed traversal of the image stack, is as produced by the original RooTrak, but with markers indicating possible backward growing roots.

**Figure 2:**
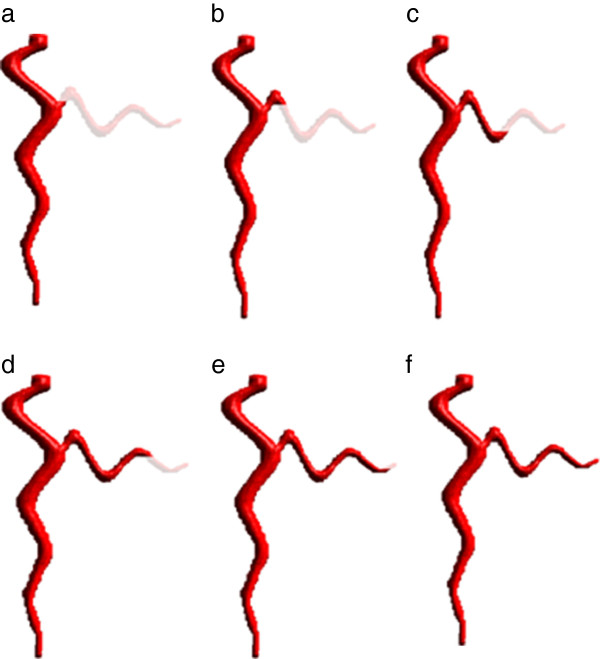
**Extraction of a simple, artificially generated, plagiotropic root by RooTrak. a**. The primary root is extracted and one upward growing section marked on the first pass through the stack. Subsequent processing focusses on the marked branch, extracting a complete description following five further tracking stages (**b**-**f**).

To complete the root system description, RooTrak then tracks upwards from each marker. Markers are examined in fixed order, from the lowest in the stack to the highest. These tracking operations may generate further markers, indicating downward growing roots that are connected to the primary root not directly, but via an upward growing root segment. When all upward growing markers have been processed, RootTrak again moves down the stack, tracking from newly reported downward markers. This process is repeated, alternating directions, until all targets are lost and no markers remain (Figure 2). Note that only the first pass must examine the entire image stack. Subsequent processing focuses on detected markers and each pass only considers images in which a previously undetected plagiotropic root is expected to be visible.

## Results and discussion

μCT data was acquired at The University of Nottingham using a Phoenix Nanotom X-ray CT scanner. Scanning resolution was 23.91 μm, X-ray voltage 110 kV and current 180 μA. 1200 projections were used in each case.

**Figure 3:**
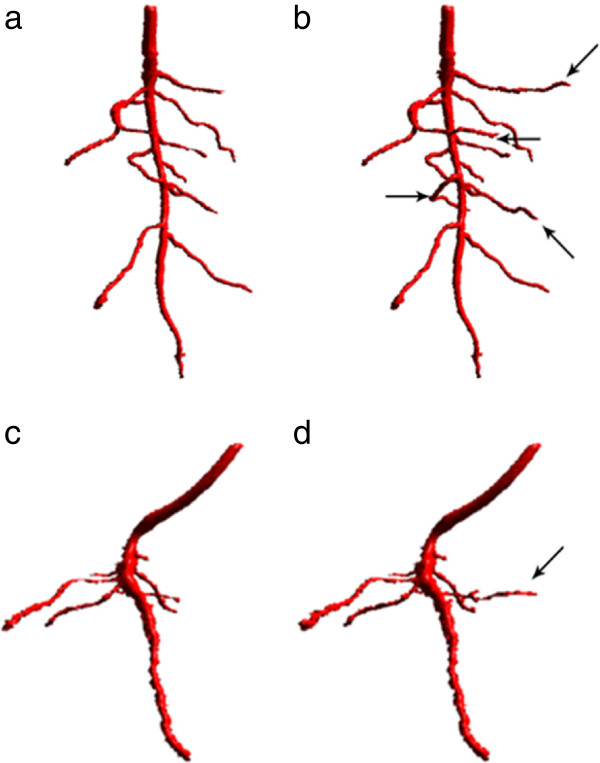
**3D visualization using volume ray-casting of data extracted by RooTrak before (a, c) and after (b, d) the extensions described here.** Arrows mark the additional roots detected.

Figure 3 shows results obtained from tomato plants (Solanum lycopersicum L) grown in clay loam (Figure 3a,b) and loamy sand (Figure 3c,d) for 10 days. A Newport series loamy sand (brown soil) and a Worcester series clay loam soil (argillic pelosol) from the University of Nottingham farm at Bunny, Nottinghamshire, UK (52.52°N, 1.07°W) were air-dried and sieved to <2 mm. These soil textures are commonly found in UK fields. Stacks of 1,388 × 1,404 × 1,313 and 1,356 × 1,352 × 1,712 pixel images were analysed to generate Figures 3a,b and c,d respectively. Figure 3a,c show root architectures recovered before, and Figure 3b,d after the extensions to RooTrak described here. Arrows indicate previously omitted root material recovered by the extended version. RooTrak requires the user to set two parameters (see [22] for details). Values of *α* = 0.606 and *β* = 0.246 were used to recover Figure 3a,b while *α* = 0.608 and *β* = 0.368 during generation of Figure 3c,d.

Table 1 compares root volume and surface area estimates computed from the data shown in Figure 1. Volume was calculated by counting the number of voxels and multiplying by voxel size cubed. Surface area is obtained by extracting the iso-surface as a mesh of triangles and summing the areas of all triangles in the mesh. There is a clear increase in identified root material. The extended RooTrak recorded an increase of c. 16.62% in root volume and c. 6.20% in surface area for Tomato 1 and c. 3.89% and c. 9.25% for Tomato 2 respectively, compared to the original version of RooTrak. It is worth noting that these plants were examined at a very early growth stage so one would expect higher detection values in a more mature plant.

**Table 1:** Measured root volume and surface area using the original RooTrak and its extension

	**RooTrak original version**		**RooTrak extended version**	
	**Tomato 1 (Figure **1**a)**	**Tomato 2 (Figure **1**c)**	**Tomato 1 (Figure **1**b)**	**Tomato 2 (Figure **1**d)**
**Volume** (mm^3^)	21.17	34.17	24.69	35.50
**Surface area** (mm^2^)	260.76	407.18	276.94	444.85
**Max. width** (mm)	50.63	54.63	50.63	55.58
**Convex hull** (mm^3^)	1623.90	3057.78	2410.18	3366.24

Besides the geometrical properties (volume and surface area) of the root system, we also wanted to assess structural differences in the descriptions produced the two different versions of RooTrak. To express and quantify the difference, we measured the maximum width as well as the volume of the convex hull enclosing the root system. The results are shown in Table 1. The maximum width was obtained by projecting all voxels to a single x-y plane and then calculating the minimum enclosing circle using Welzl’s algorithm [25]. For Tomato 1 (Figure 3a,b), the maximum width remained the same, since the additional root segments were mostly located near or around the primary root. For Tomato 2 (Figure 3c,d), on the other hand, there was a slight increase in the maximum width. A bigger difference between the two versions can be seen in the volume enclosed by the convex hull. The convex hull was computed using the QuickHull algorithm [26] in which volume is estimated using Monte Carlo Integration [27]. The reason for observing a bigger difference is that the maximum width is a one-dimensional measurement, while the convex hull, in contrast, is a function of all three dimensions. For the samples, Tomato 1 (Figure 3a,b) and Tomato 2 (Figure 3c,d) there was an increase of 48.41% and 10.08% respectively in volume.

The time needed by the original version of RooTrak to process a CT stack depends on image size, number of images, and amount of root material (see [22] for more details). Through the additional “backward-looking” step introduced here, the time required to process an image stack is doubled at best. This is because every image has to be visited at least twice; during the normal forward traversal and while looking backward (the additional step). The effort of looking for markers, however, has its advantages. Once located the extraction can be continued from each marker and stop when no objects are left to be tracked, RooTrak is not required to go through the entire image stack again in its search for opposite directed roots.

Note also that the extended RooTrak’s two stage (mark, then track) structure allows it to report the proportion of the root system which grows upward. It can also identify points at which direction of growth changes. This may be of value in itself, allowing new traits such as average length of upward/downward growing sections, angles between them etc. to be recovered. Alternatively, these changes in direction might indicate significant changes in soil properties, to which the root is responding. X-ray CT provides simultaneous imaging of both root and soil: detected changes in root direction could be used to target analysis of related soil features. Though changes in growth direction could be identified following extraction of a full geometric description of the root system architecture from RooTrak’s segmentation, the ability to recover them directly during segmentation avoids significant amounts of processing.

## Conclusions

Though the proportion of plagiotropic branches varies widely, most root systems are likely to contain some plagiotropic roots (Nakamoto, [23]). Understanding of the factors affecting angle of growth is incomplete, but there is evidence that both internal (hormonal) and external conditions (pH, temperature, oxygen and nutrient concentration) have a role to play [28]. Additional plagiotropic growth may result from disease, in particular the hairy root disease caused by Agrobacterium rhizogenes [29].

The original RooTrak [22] allows 3D descriptions of gravitropic roots growing in soil to be recovered from X-ray CT data. RooTrak adopts a visual tracking framework that is less sensitive to the natural ambiguity of X-ray attenuation data than previous approaches, and so allows a more flexible and adaptive search for roots. While previous threshold-based techniques are more rigid, and hence may not be robust in highly heterogeneous soil environments, they are usually easier to apply in higher dimensions, and capable of dealing with plagiotropism. In contrast, the greater adaptability of RooTrak’s tracking approach comes at the cost of a fixed search direction and so requires an explicit mechanism for the extraction of plagiotropic roots. The extension of RooTrak to deal with plagiotropic roots allows the tracking methodology to be applied to the full range of root architectures and will, we believe, allow higher quality root descriptions to be obtained than was previously possible. RooTrak’s tracking framework has been extended to allow both gravitropic and plagiotropic branches to be segmented and described, allowing RooTrak to produce more complete root descriptions, and so more accurate whole root system traits. Plagiotropic branches are distinguished from downward growing, gravitropic roots during the segmentation process, providing opportunities to compute new comparative (gravitropic vs plagiotropic) measures without potentially expensive, higher-level recognition of plagiotropic growth.

Comparison of the original and extended versions of RooTrak shows that for certain root system traits, results can easily be underestimated, even if only a small fraction of the root system is missing. It is important to recover as much root material as possible when estimating root system characteristics. This is especially the case for plant species having a large number of plagiotropic roots, for which the error is not negligible.

## Availability and requirements

RooTrak is open source and available from SourceForge via http://www.rootrak.net. The tool is written in C++ and includes Visual Studio 2008 project files to compile for Windows. The Qt 4.8 framework is required for the compilation of the source code. RooTrak incorporates a volume rendering tool which displays the root system using GLSL, and so requires a GLSL compliant graphics card.

## Competing interests

The authors declare that they have no competing interests.

## Authors’ contributions

SM designed and implemented the extended RooTrak software. SZ and ST provided advice on plant preparation and growth and CS captured the image data. SJM, MJB and TPP devised the research, provided analysis and contributed to the article. All authors read and approved the final manuscript.
